# Effects of Different Dietary Selenium Sources on Antioxidant Enzyme Activities and Selected Hematological Parameters in Weaned Piglets

**DOI:** 10.3390/ani16111700

**Published:** 2026-06-01

**Authors:** Kristina Gvozdanović, Jakov Jurčević, Zlata Kralik, Ivona Djurkin Kušec, Goran Kušec, Mislav Đidara, Marcela Šperanda, Katarina Marić, Vladimir Margeta

**Affiliations:** 1 Faculty of Agrobiotechnical Sciences Osijek, Department for Animal Science and Biotechnology, 31000 Osijek, Croatia; zkralik@fazos.hr (Z.K.); idurkin@fazos.hr (I.D.K.); gkusec@fazos.hr (G.K.); mislav.didara@fazos.hr (M.Đ.); msperan@fazos.hr (M.Š.); vmargeta@fazos.hr (V.M.); 2 Farmdizajn d.o.o., Ulica Hrvatske Republike 17B, 31000 Osijek, Croatia; jj@farmdizajn.com; 3 Belje Plus d.o.o., Svetog Ivana Krstitelja 1a, 31326 Darda, Croatia; kamaric@fazos.hr

**Keywords:** piglets, weaning, selenium, hematological parameters, thiobarbituric acid reactive substances

## Abstract

The aim of this study was to investigate the effects of different selenium sources on hematological parameters and antioxidant enzyme activities in weaned piglets. Two hundred crossbred (Duroc × Landrace × Yorkshire) piglets were divided into four groups (n = 50) supplemented with different forms of selenium: control group (K; standard diet without selenium supplementation), organic selenium (P1), selenium from biofortified feeds (P2), and nano-selenium (P3). Blood samples were collected on days 0, 22, and 45 post-weaning. A significant effect of selenium supplementation was found for mean corpuscular hemoglobin concentration (*p* = 0.029) and platelet count (*p* = 0.049). Selenium source affected glutathione peroxidase in plasma (*p* = 0.003) and erythrocytes (*p* = 0.001), as well as carbonyl proteins (*p* = 0.001). At the same time, treatment × sampling time interactions were significant for glutathione peroxidase in plasma (*p* = 0.001) and carbonyl proteins (*p* = 0.001). Nano-selenium showed relatively consistent antioxidant enzyme activity over time. Thiobarbituric acid reactive substances values in different muscles increased significantly after 7 days in all analyzed muscles. A treatment effect was observed only in *m. triceps femoris* (*p* = 0.048), while a significant treatment × storage interaction was detected in *m. intercostales sternales* (*p* = 0.022). Although the time after weaning had a strong influence on the hematological parameters, selenium supplementation mainly modulated antioxidant parameters, while its effect on lipid oxidation was muscle-dependent.

## Simple Summary

Weaning is one of the most stressful phases in pig production. The stressors to which piglets are exposed affect their health status and ability to cope with oxidative stress. Selenium, an important dietary microelement, plays a key role in protecting cells from oxidative damage. This research examined the influence of different dietary sources of selenium on hematological indicators, antioxidant status, and oxidative stability of muscle tissue in weaned piglets. The results showed that the post-weaning period strongly influenced most blood indicators. The addition of selenium, especially in its nanoform, affected indicators of antioxidant enzyme responses. Lipid oxidation in muscle tissue depended mainly on the duration of storage, while the effect of selenium was limited and dependent on the type of muscle. These findings provide insights into how to design nutritional strategies that support antioxidant protection in piglets after weaning.

## 1. Introduction

Selenium (Se) is an essential trace element important for maintaining homeostasis in pigs [1,2]. Its significance is evident in its role in antioxidant defense, inflammation control, and immune function [3]. To fulfill its biological function, Se must be supplied in pig diets, mainly in organic (selenomethionine and selenocysteine) and inorganic (selenate and selenite) forms [4,5]. Se deficiency can lead to health problems, such as muscle disorders, reduced fertility, weakened immune function, and impaired growth [6,7]. According to FDA standards, Se supplementation in complete feed is limited to 0.3 mg/kg [8]. Nutritional requirements for Se range from 0.3 mg/kg for weaned piglets to 0.15 mg/kg for fattening pigs [9]. Sources of Se in nature and food are limited, as most soils are low in this nutrient. Therefore, in recent years, soil biofortification with Se has been implemented, thereby significantly increasing its content in plants, cereals, and food products [10]. Currently, in most cases, biofortification uses inorganic Se, which has proven to be a lower-input solution than biofortification with organic Se, and the effectiveness is almost identical, as plants convert inorganic Se into organic forms during metabolic transformations, making it available to animals [11]. Biofortified feed is a natural source of Se, primarily found in organic forms. Nano-Se, by contrast, is an engineered form with very small particles that may offer improved bioavailability and antioxidant activity [12,13]. In all pig categories, Se supplementation is widely applied in reproductive sows, but its role is also important in the nutrition of weaned piglets. Weaning is one of the most stressful phases [14]. The period immediately after weaning is characterized by immunological changes, such as upregulation of inflammatory cytokines and increased concentrations of acute-phase proteins. The effectiveness of Se supplementation in the nutrition of weaned piglets is important for maintaining homeostasis in several metabolic processes and for protecting proteins, lipids, and cell membranes from oxidative damage [15,16]. Surai [15] states that the main advantage of organic Se in the nutrition of weaned piglets is its role in creating Se reserves in the body. Under stress, when the need for antioxidants increases but feed intake usually decreases, Se reserves accumulated in tissues may be crucial for pigs’ resistance to the harmful effects of free radicals and toxic metabolic products. It has been shown that the use of organic Se in piglet nutrition can improve their antioxidant defense, potentially enhancing the animals’ ability to adapt to various stresses [17,18,19]. However, the effect of Se supplementation depends on the duration of supplementation and the physiological status of the pigs [18,19].

Se is included in pig nutrition to prevent diseases caused by Se deficiency [6,7] and to combat various infections due to its immunomodulatory characteristics. Organic Se in sow diets, compared to inorganic forms, significantly improves antioxidant status in weaned piglets and thyroid metabolism. Recent studies have also shown that organic Se, compared with inorganic Se, positively affects litter weight, average piglet weight, and daily gain from birth to weaning [20]. Organic Se supplementation has also been reported to significantly improve growth performance, antioxidant capacity, and plasma Se content in weaned piglets [4,21]. Organic Se was effective in reducing inflammation and oxidative stress in piglets orally infected with *Salmonella typhimurium* by inducing lymphocyte activity and the expression of antioxidant enzymes [2]. Se also has the potential to alleviate immunosuppression caused by the mycotoxin deoxynivalenol in piglets [22]. Se in the form of nanoparticles is increasingly used as a new source of Se in piglet nutrition [12]. Numerous studies indicate that nano-Se enhances intestinal antioxidant status and improves the activity of key antioxidant enzymes [13,23]. In a study by Qiao et al. [13], supplementation with Se nanoparticles significantly improved various antioxidant parameters in piglets. Specifically, there was an increase in jejunal Se levels, as well as improved jejunal activities of total antioxidant capacity, catalase, total superoxide dismutase, glutathione peroxidase, and thioredoxin reductase. Since the post-weaning period is marked by increased oxidative stress, immune challenges, and often reduced feed intake, Se sources such as biofortified feed and nano-Se may support antioxidant-related responses during this sensitive stage. In such cases, Se sources may be relevant for maintaining antioxidant enzyme-related responses even when food intake is low. From a practical point of view, nano-Se and Se-biofortified feed sources could be of interest in commercial post-weaning diets, since this period is characterized by reduced feed intake, oxidative stress, and intestinal adaptation. These sources may be an alternative strategy to support antioxidant and gut-related responses in piglets, but their effects under commercial conditions require further evaluation. However, there is limited data comparing biofortified Se and nano-Se with other dietary Se sources in weaned piglets, especially regarding their effects on hematological parameters, antioxidant enzyme activities, and the oxidative stability of muscle. For this reason, evaluating these forms of Se in the context of post-weaning adaptation is particularly relevant.

The aim of this study was to evaluate the effects of dietary biofortified and nano-Se supplementation on hematological parameters, antioxidant enzyme activities, and lipid oxidation in weaned piglets during the post-weaning period.

## 2. Materials and Methods

The experimental protocol was approved by the Bioethics Committee of the Faculty of Agrobiotechnical Sciences Osijek, and all procedures were performed in accordance with the Croatian Animal Welfare Act and other legal acts regulating animal husbandry and welfare.

### 2.1. Animals, Feeding, and Housing System

A total of 200 crossbred (Duroc × Landrace × Yorkshire) (100 castrated males and 100 females) piglets, weaned at 28 days of age, and all from the same commercial strain (Pig Improvement Company, Hendersonville, TN, USA), were used. Piglets were assigned in a completely randomized design and distributed equally according to the feeding treatment into four groups, with the experimental group consisting of 50 piglets. The treatments were distributed as follows: control group, which received a standard corn–soybean basal diet without additional Se supplementation (K, n = 50); basal diet supplemented with organic Se (P1, n = 50); basal diet supplemented with Se from a biofortified feed (P2, n = 50); and basal diet supplemented with nano-Se (P3, n = 50). All groups received the same basal corn–soybean diet (Table 1). The control group received a standard corn–soybean basal diet without additional Se supplementation. Experimental diets were formulated to provide a total Se concentration of 0.3 mg Se/kg, provided either as organic Se (P1), Se from biofortified feed (P2), or nano-Se (P3). The composition and chemical analysis of the basic feeding mixture are presented in Table 1. A single-phase basal diet was used throughout the entire post-weaning experimental period for all treatment groups. Corn enrichment with Se was conducted by soil biofortification using sodium selenate (Na_2_SeO_4_) at a rate of 35 g/ha. Se-biofortified corn was produced for experimental purposes. The analyzed Se concentration in the biofortified corn was 0.266 mg/kg. In the P2 group, biofortified corn replaced the same proportion of conventional corn feed flour in the basal diet formulation. Remaining ingredients and nutritional composition of the diets remained identical across treatments. Nano-Se (FRA Easy Selenium Dry, FRAmelco^®^, Raamsdonksveer, The Netherlands), produced by chemical synthesis, was added directly to the basal diet as a separate Se source. According to the manufacturer’s specification, the nano-Se powder had an average particle size of 10–45 nm, a specific surface area of approximately 30–50 m^2^/g, and a purity of 99.9%. The experimental Se sources added to the basal diet in P1 were Se yeast (total Se 1000 mg/kg, organic Se, Sel-Plex; Alltech, South Brookings, SD, USA).

The piglets were weighed three times during the experimental period: at weaning (day 0), 22 and 45 days after weaning by a commercial pig weighing scale (Pig Scale WA-P1; Elicom Electronic Ltd., Silistra, Bulgaria). Average daily gain (ADG) was calculated for the corresponding experimental periods from body weight data. At weaning (28 days of age), piglets were randomly assigned to the feeding treatments. Piglets were housed in nursery cages with a slatted floor (0.4 m^2^ *per* piglet), with 10 piglets *per* pen, under controlled environmental conditions. Each pen contained a balanced number of castrated males and females. Sex distribution was balanced within pens during group assignment. The feeding treatment was applied at the pen level, where 50 piglets from each feeding group were equally distributed (10 piglets *per* pen). All piglets were individually identified using ear tags. For laboratory analyses, individual piglets were randomly selected within each feeding group and considered observational units. For blood analyses, including hematological and antioxidant parameters, 10 piglets per treatment group were sampled at each sampling time. The same piglets were sampled repeatedly on days 0, 22, and 45 after weaning. During the research, the piglets received feed and water ad libitum from automatic feeders and drinkers. At the end of the experimental period, 45 days after weaning, the piglets were transported to a nearby commercial abattoir where they were slaughtered according to the conventional procedure that included stunning with CO_2_. For muscle tissue analysis, piglets were randomly selected within each treatment group among clinically healthy animals. Muscle tissue samples were collected from 10 piglets per treatment group. A single sample was collected from each piglet and divided into two parts. Following collection, fresh muscle samples were stored on ice and transported to the Laboratory at the Faculty of Agrobiotechnical Sciences. The first part was analyzed for TBARS within 24 h of sample collection (day 0). The second part of each sample was stored at 4 °C under refrigerated conditions for 7 days before analysis.

### 2.2. Chemical Analysis of Feed

The basic chemical composition of feeds by group was analyzed in two parallel replicates using standard methods [24]. Metabolizable energy was estimated based on the diet formulation and tabulated ingredient values and was not directly determined by chemical analysis. Crude protein concentration was estimated from nitrogen content using the Kjeldahl method [25]. The Weende method was used to analyze crude fiber concentration [26]. The Extraction System B-811 (Büchi, Flawil, Switzerland) was used to determine crude fat concentration [27].

The Se content of the final feed mixtures was analyzed in two parallel samples. The feed samples for Se determination were digested with HNO_3_ and H_2_O_2_ in a microwave oven (MARS 6; CEM Corporation, Matthews, NC, USA) for 25 min. After digestion, HCl was added to the samples. The prepared samples were dried at 90 °C and then cooled to room temperature. The Se content was measured using inductively coupled plasma optical emission spectrometry (ICP-OES; Optima 2100 DV, PerkinElmer Inc., Waltham, MA, USA; [28]).

### 2.3. Hematological Analysis

Blood from the jugular vein was collected on days 0, 22, and 45 using the Vacutainer system in lithium heparin anticoagulant tubes (Becton, Dickinson, Plymouth, UK). The 2 mL of blood samples were centrifuged at 1.500× *g* for 10 min at 4 °C, and the blood plasma was separated and frozen at −80 °C. Hematological parameters (white blood cell count (WBC), red blood cell count (RBC), hemoglobin concentration (HGB), hematocrit (HCT), mean corpuscular volume (MCV), mean corpuscular hemoglobin (MCH), mean corpuscular hemoglobin concentration (MCHC), platelet count (PLT)) were analyzed using the analyzer Poch-100iV Diff (Sysmex Corporation, Kobe, Japan) according to the manufacturer’s instructions.

### 2.4. Assessment of Antioxidant Enzyme Activities and Lipid Oxidation

The activity of glutathione peroxidase (GPX) in plasma was determined using a Ransel^®^ kit (RANDOX Ransel kit RS50; Randox Laboratories Ltd., Crumlin, UK). Superoxide dismutase (SOD) activity was measured using a Ransod^®^ kit (RANSOD SD 125; Randox Laboratories Ltd., Crumlin, UK). The serum catalase (CAT) activity was determined using the method described by Aebi et al. [29]. All analyses were performed using an automatic Beckman Coulter AU 400 analyzer (intra-assay CV < 5% and inter-assay CV < 10%, Beckman Coulter Inc., Brea, CA, USA).

Lipid oxidation was quantified by measuring thiobarbituric acid reactive substances (TBARS) content in samples of *m. triceps femoris*, *m. longissimus dorsi*, and *m. intercostales sternales*. Analyses were performed on fresh meat samples (day 0) and after 7 days of refrigerated storage at 4 °C. The samples were prepared as follows: 4 g of muscle tissue was weighed into a test tube, and 10% trichloroacetic acid was added; the mixture was homogenized and centrifuged at 5500× *g* for 15 min at 4 °C. Then, 2.5 mL of the supernatant was pipetted, and 1.5 mL of thiobarbituric acid solution (pH 2.5) was added. The test tubes were closed and placed in a water bath at 95 °C for 30 min. After cooling, distilled water was added, and the mixture was centrifuged at 5500× *g* for 15 min at 4 °C. The content of the colored product formed by the reaction of lipid peroxidation products with thiobarbituric acid was measured spectrophotometrically at 532 nm (intra-assay CV 4% and inter-assay CV 6–7%). TBARS values were quantified using a standard calibration curve prepared with malondialdehyde tetrabutylammonium salt (Sigma-Aldrich, Buchs, Switzerland) and expressed as μg MDA/g muscle tissue.

### 2.5. Carbonyl Proteins

After weighing and homogenizing the tissue in 0.1 M phosphate buffer (1:4), the sample was centrifuged. The supernatant was mixed with 30% TCA (trichloroacetic acid), centrifuged again, and the resulting precipitate was used to determine the concentration of carbonyl proteins (CP). DNPH (dinitrophenylhydrazine) solution is added to the precipitate, which is then vortexed and incubated for 1 h at room temperature in the dark. Centrifugation is repeated, and the precipitate is washed three times with a mixture of ethanol and ethyl acetate. The precipitate is then dissolved in guanidine-HCl, vortexed, and centrifuged. The amount of CP in the supernatant is measured spectrophotometrically at 370 nm (intra-assay CV < 6% and inter-assay CV < 8%). Protein concentration in tissue homogenates was determined by the Bradford method [30], using bovine serum albumin (BSA) as the standard. Absorbance was measured at 595 nm, and protein concentration was calculated from a calibration curve. Protein determination was performed on an aliquot of homogenate before DNPH derivatization to avoid reagent interference. Values are expressed as nmol CP *per* mg protein.

### 2.6. Statistical Analysis

Statistical analyses were conducted using the dplyr package [31] and car [32] in the R environment [33]. Repeated-measures ANOVA was performed using the ez [34] and afex packages [35], while linear mixed-effects models were fitted using the lmerTest package [36]. The dataset was checked for outlier values, which were excluded when identified as biologically implausible values or as analytical or recording errors. No missing data were present in the final dataset used for statistical analysis. The Levene test and the Shapiro–Wilk test were used to assess data distribution and homogeneity of variance. Assumptions for repeated-measures ANOVA were evaluated for all variables included in the repeated-measures analyses. All numerical data are presented as mean values with the standard error of the mean (SEM). A two-way analysis of variance with repeated measures over time was used to evaluate the effects of feeding treatment, time post-weaning, and their interaction on hematological parameters and antioxidant enzyme activities. Repeated measurements were accounted for by treating sampling time as the within-subject factor. Feeding treatment was analyzed as a between-subject factor and time as a within-subject factor. The sphericity assumption was assessed using Mauchly’s test to evaluate the covariance structure of repeated measurements. When the assumption was violated (*p* < 0.05), the Greenhouse–Geisser correction was applied to adjust the degrees of freedom. When significant main effects or interactions were detected, Tukey-adjusted post hoc comparisons were performed to identify differences between treatments, sampling times, or their interactions.

TBARS data were analyzed using a linear mixed-effects model that considered feeding treatment, storage time, and their interaction as fixed effects. An individual piglet was included as a random effect to account for repeated measurements across storage time. Statistical significance was assessed using Type III ANOVA with the Satterthwaite approximation of degrees of freedom. Tukey-adjusted *post hoc* comparisons based on estimated marginal means were performed when significant effects were detected (*p* < 0.05). Individual piglets were considered the observational units for statistical analyses. Sex and initial body weight were not included as covariates, as animals were balanced during group assignment. Although treatments were applied at the pen level, recorded pen identification was not available for the individually sampled piglets; therefore, the pen could not be included as a random effect in the statistical model. This was considered a limitation of the statistical approach, and treatment-related effects were interpreted with caution.

## 3. Results

The body weights of the piglets depending on Se supplementation were not significantly different (*p* > 0.05). Mean body weights (±SEM) at day 0, day 22, and day 45 were 6.45 ± 0.12, 11.85 ± 0.34, and 21.57 ± 0.85 kg in the K group; 6.40 ± 0.12, 11.99 ± 0.41, and 20.80 ± 0.48 kg in the P1 group; 6.60 ± 0.12, 12.65 ± 0.93, and 21.03 ± 1.62 kg in the P2 group; and 6.40 ± 0.10, 12.07 ± 0.48, and 21.41 ± 1.04 kg in the P3 group, respectively. Body weight did not differ significantly among treatments during the post-weaning period (*p* > 0.05) (Supplementary Table S1). The effects of Se supplementation from different dietary sources on the hematological parameters of piglets at days 0, 22, and 45 post-weaning are shown in Table 2. The results show no significant effect of Se supplementation between the analyzed groups for most parameters (*p* > 0.05). A treatment effect was observed only for MCHC (*p* = 0.029) and PLT (*p* = 0.049). Tukey-adjusted post hoc comparisons showed that the P3 group of piglets had significantly lower MCHC values compared to the P2 group (*p* = 0.020). No other pairwise differences were statistically significant (*p* > 0.05). Post hoc analysis showed that PLT levels were significantly higher in the group supplemented with nano-Se compared to the control group (*p* = 0.043). No other pairwise differences were statistically significant (*p* > 0.05).

No significant differences were found for the interaction between Se supplementation and weaning days in any of the hematological parameters analyzed (*p* > 0.05). The effect of sampling time after weaning was observed in all analyzed parameters except for PLT (*p* > 0.05). Considering the overall effect of sampling time, WBC levels were significantly higher on day 22 compared with day 0 (*p* = 5.8 × 10^−9^) and on day 45 compared with day 0 (*p* = 0.00076), while no significant difference was detected between days 22 and 45 (*p* = 0.102). RBC levels were significantly higher on day 45 compared to day 0 (*p* = 0.00027) and day 22 (*p* = 0.039), and were also higher on day 22 compared to day 0 (*p* = 0.045).

Although numerical differences were observed among treatments, no significant treatment effect was detected for HGB, HCT, MCV, and MCH (*p* > 0.05). HGB levels were significantly lower on day 22 (*p* = 1.4 × 10^−9^) and day 45 (*p* = 7.4 × 10^−5^) compared to day 0, with no significant difference between days 22 and 45 (*p* = 0.55). For HCT levels, significantly lower values were reported on day 22 than on day 0 (*p* = 0.0009), whereas no significant differences were found between the other time points (*p* > 0.05).

The antioxidant enzyme activities in the blood of piglets at different days post-weaning are presented in Table 3. A significant treatment effect was observed for glutathione peroxidase in plasma (GPX_P_) (*p* = 0.003), glutathione peroxidase in erythrocytes (GPX_E_) (*p* = 0.001), and carbonyl proteins (*p* < 0.001), indicating that the values differed among dietary treatments. No differences were observed for other analyzed parameters (*p* > 0.05). The highest GPX_P_ levels in the control group were observed at day 45. Values recorded on day 0 were considered baseline values and not treatment responses. Therefore, differences observed among treatment groups at day 0 were interpreted as baseline variability. The time post-weaning significantly affected all analyzed parameters (*p* = 0.001).

The significant interaction effect between treatment and post-weaning days was observed for GPX_P_ (*p* = 0.001), SOD_E_ (*p* = 0.038), catalase (*p* = 0.046), and carbonyl levels (*p* = 0.001). GPX_P_ activity on the 45th day was significantly higher in the control group compared to P1 (*p* = 0.00008) and P3 (*p* = 0.00001). Catalase activity on day 45 in the control group differed significantly compared to P1 (*p* = 0.011) and P3 (*p* = 0.00002). Levels of carbonyl proteins were the lowest in the K group at day 45 and differed significantly from P2 (*p* = 0.0168) and P3 (*p* = 0.0022).

The effect of different dietary Se sources on lipid oxidation (TBARS) in *m. triceps femoris* (TB), *m. longissimus dorsi* (MLD), and *m. intercostales sternales* (MIS) was measured in fresh meat (day 0) and after 7 days of refrigerated storage (Table 4). The results show a significant effect of Se supplementation on TBARS levels in the TB muscle (*p* = 0.048), while no significant effect was observed in MLD and MIS muscles (*p* > 0.05). Storage time significantly affected TBARS values in all analyzed muscles (*p* = 0.001). An interaction effect was observed only for TBARS measured in MIS muscle (*p* = 0.022).

## 4. Discussion

The effect of Se supplementation on hematological parameters (Table 2) was found only for MCHC and PLT levels, while time after weaning significantly affected most hematological parameters of piglets except PLT. The levels of leukocytes increased in all groups between weaning (day 0) and 22 days after weaning, ranging from 13.40–16.90 ×10^9^/L at the beginning to 20.53–23.60 ×10^9^/L on day 22. After that, a slight decline and stabilization were recorded on day 45 post-weaning. The observed changes in hematological parameters are probably related to the physiological adaptation of piglets to the stress that occurs during the weaning phase [14,37]. The results indicated that Se supplementation significantly affected MCHC and PLT, but time remained the dominant factor influencing hematological parameters during the post-weaning period. Because body weight and ADG did not differ significantly among treatments, the observed hematological and antioxidant enzyme responses are unlikely to be explained by major differences in growth rate. However, effects associated with nutritional treatments should be interpreted with caution because the treatments were applied at the pen level. As pen identification for individually sampled piglets was not available, the pen could not be included as a random effect in the statistical model.

Liu et al. [21] and Lv et al. [2] reported that the leukocyte response of piglets after weaning changes due to the stress experienced during this production phase. The increase in leukocytes from weaning to day 21 post-weaning represents part of the organism’s adaptive response to stress immediately after weaning, regardless of the Se source. The total leukocyte levels observed in our study are within the range reported by Zhang et al. [38], ranging from 7.18 to 24.52 × 10^9^/L. In contrast, the WBC values recorded in piglets immediately after weaning (day 0) are higher than those reported by Rolinec et al. [39], which were 10.82 × 10^9^/L. In our study, RBC levels increased over time after weaning, while HGB levels were highest immediately after weaning (day 0). Friendship et al. [40] and Rolinec et al. [39] have shown that, due to metabolic processes and changes in the hematological profile during growth, RBC values increase with the age of piglets.

Zhang et al. [38] also found that changes in hematological parameters in short-term studies, especially RBC and WBC, are not necessarily significant. In addition to the fact that these changes depend on the source of Se in piglet diets, they also depend on the duration of the feeding and production experiment. In contrast to our study, Kyoung et al. [41] reported that RBC levels in the control group of piglets fed a corn and soybean meal-based diet were higher (6.88 × 10^6^/μL 42 days after weaning and 8.05 × 10^6^/μL 14 days after weaning, respectively) compared to RBC levels in piglets that received organic and inorganic Se in their diets. Furthermore, the authors reported hemoglobin concentrations ranging from 112.2 to 137.7 g/L, which are higher than the HGB levels observed in our study. These differences may be attributed to the duration of the feeding treatment, as well as the nutritional and physiological status during the post-weaning period. The HCT values in our study ranged from 35.00% to 38.00% and are consistent with the results of Zhang et al. [42]. The authors state that piglets weaned at 28 days of age and supplemented with organic Se had plasma HCT values of 37.6% to 41.4%. Furthermore, Kyoung et al. [41] reported different HCT values between piglets supplemented with organic and inorganic Se in their diets and those in the control group, but these differences were not statistically significant. Piglets that received Se supplementation at 7, 14, and 22 days after weaning had HCT values of 38.27%, 39.48%, and 27.30%, while the control group recorded values of 38.67%, 41.40%, and 30.27%. A similar pattern was observed in our study for MCV and MCH levels of the analyzed groups of piglets. Piglets undergo intense physiological changes during the post-weaning period. Hematological parameters, including hemoglobin, are known to change with age and development [38,40]. Red blood cell dynamics and hemoglobin synthesis are influenced by changes in plasma volume and immune activity [43]. Se, as a component of selenoproteins, has been reported to contribute to erythrocyte membrane stability and the reduction in oxidative damage [44]. However, these mechanisms were not directly evaluated here, and hematological changes should therefore be interpreted with caution. Thus, the effect of Se supplementation on hemoglobin changes is more likely related to physiological adaptation during the post-weaning period. According to Zhang et al. [38], reference values for MCV and MCH are 47.28–62.74 fL and 13.90–18.81 pg, respectively. Kyoung et al. [41] reported lower MCV values of the control group of piglets and in the group supplemented with the organic form of Se compared to our results.

Se supplementation effects were observed for MCHC and PLT levels of the analyzed piglet groups. As no significant treatment × time interaction was detected, these differences reflect overall treatment effects rather than changes at specific time points. All recorded MCHC values were within the reference range for piglets immediately after weaning (day 0), which, according to Zhang et al. [38], is 280.50–313.80 g/L, although there were statistically significant differences between treatments. Similar changes in erythrocyte indices were also observed in the study by Zhang et al. [42], where linear and quadratic effects of Se supplementation influenced MCH and MCHC values. In the study by Kyoung et al. [41], MCHC in the control group of piglets and the group supplemented with organic Se ranged from 328.00 g/L and 330.00 g/L seven days post-weaning to 412.20 g/L and 412.12 g/L forty-two days after weaning, which is higher than our results. The authors state that Se can modulate the proportion of immature erythrocytes and hemoglobin dynamics, especially under conditions of metabolic and oxidative stress. Se supplementation had a significant effect on PLT levels, while the post-weaning period did not significantly affect the level of PLT in plasma. PLT values for all analyzed groups ranged within a wide physiological range for piglets after weaning (38.74–656.85 × 10^9^/L; [38]). Hematological responses to treatment were limited to MCHC and PLT, although Se source had a statistically significant but biologically limited effect on PLT. Thus, these results do not allow conclusions on systemic physiological effects associated with Se bioavailability under the present experimental conditions [12,13,45]. Kyoung et al. [41] reported higher PLT values than those found in our research. The authors observed that PLT levels were lowest on the 42nd day after weaning in the piglets’ control group and highest in the group supplemented with organic and inorganic Se. In contrast, Zhang et al. [42] reported lower PLT levels of piglets supplemented with Se-yeast and DL-selenomethionine, with no significant differences between treatments.

Weaning is the most challenging phase in pig production [46]. The addition of Se to the diets of weaned piglets increases the activity of antioxidant enzymes [45], has positive effects on immune function [2], and improves health status [18]. Superoxide dismutase (SOD) and glutathione peroxidase (GPX) are considered antioxidant enzymes that protect cells from injury caused by O_2_^−^ [47,48,49]. The results in Table 3 show that both time after weaning and Se supplementation were associated with changes in the antioxidant enzyme activities of piglets. The decline in SOD and CAT activity immediately after weaning (day 0) up to 45 days suggests an increasing oxidative challenge during the post-weaning period. A study by Qiao et al. [13], in which sodium Se was replaced with biogenic Se nanoparticles in diets for early weaned piglets, showed that Se nanoparticles affected Se levels and antioxidant enzyme activities. As a result, SOD activity was preserved, and GPX activity was enhanced compared to the inorganic Se source. These findings are consistent with the results of our study, which showed a decrease in SOD activity (17–22%) in Se-supplemented groups compared to the control group (30%). Similar changes in antioxidant enzyme activity have been reported by Lv et al. [2], Zhang et al. [42], and Rao et al. [45]. Liu et al. [21] found that supplementation with organic Se significantly increased the activities of SOD and GPX in serum. The authors reported that the Se-supplemented groups had higher activities of SOD and GPX compared to the control group. Lv et al. [2] also investigated the effect of supplementing different forms of Se in the diets of weaned piglets. Similar to our results, Lv et al. [2] found decreases in SOD and GPX activity during the early phase and higher activity in piglets supplemented with Se. GPX is a Se-dependent enzyme, and it reflects the availability of Se in the body [3,50,51]. Furthermore, an interaction effect was observed between supplementation with different forms of Se and the time period after weaning on GPX levels in piglet plasma. In the control group, which did not receive Se supplementation in the diet, GPX activity increased from weaning to 45 days post-weaning, which may indicate an adaptive or compensatory enzyme response during the post-weaning period rather than improved antioxidant protection. In contrast, the groups that received Se in their diets showed lower enzyme activity over time. Notably, a relatively consistent temporal pattern of GPX activity was observed in the nano-Se-supplemented group, consistent with the findings of Qiao et al. [13]. The authors suggested that nano-Se, possibly due to its specific physicochemical characteristics, may influence antioxidant enzyme responses related to reactive oxygen species regulation compared with other forms of Se. Several studies have shown the effect of nano-Se on GPx activity compared to other dietary sources of Se [12,13,52]. Nano-Se particles may have improved absorption and tissue distribution compared to other Se forms, which may facilitate their incorporation into selenoproteins [12]. However, differences in Se bioavailability, Se status, or tissue distribution cannot be directly confirmed in the present study, as plasma or tissue Se concentrations were not measured. Analyses on other animal species have shown that Se nanoparticles exhibit different antioxidant-related responses compared with organic and inorganic Se sources [51,53]. Araujo et al. [53] confirmed the greater effectiveness of nano-Se supplementation in preventing oxidative stress and enhancing antioxidant activity in tissues compared to organic and inorganic Se sources. Encapsulation and particle size may influence the biological activity of nano-Se and its role in selenoprotein-related processes. Wang et al. [52] state that nano-Se provides stable Se availability for the incorporation of selenocysteine into the enzyme’s active site and, over time, stimulates GPx activity.

The relatively limited effect of the experimental Se treatments can be explained by the fact that this level represents the recommended nutritional, but not pharmacological, dose. The absence of a separate inorganic Se treatment should be considered when comparing the obtained results with studies based on conventional inorganic Se supplementation. When the basic diet contains lower amounts of Se, additional supplementation does not have a linear effect but remains within physiological levels. A similar pattern has been observed for antioxidant activity, where GPx activity depends on the Se dose in the diet but does not increase linearly [50]. Additionally, nano-Se and organic forms of Se may have different biological effects compared with inorganic sources at the same dose of 0.3 mg/kg [12,45,54]. Therefore, the results of this study showed statistically detectable but moderate changes in hematological and antioxidant parameters and lipid oxidation, but did not provide direct evidence of improved systemic antioxidant protection. Furthermore, the results indicate that Se supplementation at a dose of 0.3 mg/kg, regardless of the chemical form, modulated antioxidant enzyme responses during the post-weaning period. CAT activity decreased during the post-weaning period in all groups of piglets. The results indicate that Se supplementation was not sufficient to preserve CAT activity in the later stages after weaning. Lv et al. [2] and Liu et al. [18] showed that CAT activity under stress conditions was higher in piglets supplemented with the organic form of Se compared to piglets supplemented with inorganic Se. The authors confirmed that the source of Se impacts CAT activity, with the organic form of Se being more effective. Also, they observed changes in inflammatory markers and confirmed the connection between antioxidants and immune regulation during the post-weaning period. Carbonyl proteins are indicators of oxidative stress in the body. Their increase during the later stages of weaning occurs as a consequence of changes associated with physiological stress [14,41,46], nutritional changes [19,55], and the antioxidant system [56,57]. Although baseline carbonyl levels were lower in piglets assigned to the nano-Se and biofortified Se groups, carbonyl levels were elevated in the later stage after weaning (day 45) in all groups analyzed. Because some antioxidant-related variables were already different between treatment groups at baseline (day 0), further comparisons between treatment groups should be performed with caution. These later differences may partly reflect initial between-group variability rather than treatment effects alone. These results suggest that Se supplementation did not prevent the increase in carbonyl protein levels during the later post-weaning period [41,56]. The increase in carbonyl concentration was associated with the decrease in catalase activity and the increase in GPX activity observed in the control group of piglets. This pattern suggests that changes in catalase and glutathione peroxidase activity are associated with increased oxidative stress during the post-weaning period, with the increased GPX activity in the control group reflecting an adaptive response to disturbed redox balance rather than improved antioxidant protection.

Thiobarbituric acid reactive substances (TBARS) are used as biomarkers for lipid peroxidation, typically indicating oxidative damage to lipids and cell membranes [58,59]. As Se is an essential component of antioxidant enzymes, its role in the oxidative stability of tissues is particularly important. Research has shown that the effect of Se supplementation on TBARS values in tissues depends on several factors, such as the physiological status of the animal [60], the duration of storage [61,62], and the form and concentration of Se in animal diets [10,45,54]. Our results showed that TBARS values were influenced by storage time, while the effect of Se supplementation was limited and muscle-specific, with a statistically significant effect observed only in the *m. triceps femoris* (TB), while a significant treatment × storage interaction was detected in *m. intercostales sternales* (MIS) (Table 4). In the present study, the effect of Se supplementation on TBARS was not consistent among the different muscles, since a significant treatment effect was found only in *m. triceps femoris*, while the storage time significantly increased the TBARS values in all the muscles analyzed. This suggests that the response to Se supplementation was lower than the effect of storage and was dependent on the muscle analyzed. Differences in oxidative stability between pork muscles have previously been associated with muscle-dependent differences in fatty acid composition, total iron content, and antioxidant capacity [63,64]. Similarly, the studies of Lv et al. [2] and Rao et al. [45] did not find a significant influence of Se supplementation in the diets of weaned piglets on TBARS values in tissue. Interestingly, groups of piglets supplemented with organic Se (P1) and Se from biofortified feed (P2) showed a numerically lower increase in TBARS values compared to the control group. Cao et al. [4] demonstrated the influence of selenomethionine supplementation on the antioxidant status of weaned piglets. The authors found that DL-selenomethionine supplementation improved antioxidant status in piglets and reduced muscle MDA levels. However, differences between organic and inorganic sources were small and not statistically significant. Furthermore, they concluded that more bioavailable forms of Se may contribute to a more favorable oxidative balance, but their effect on post-mortem lipid oxidation remains limited. A significant interaction between Se supplementation and storage time was found only in *m. intercostales sternales*. These results indicate that the dynamics of TBARS increase during storage differed depending on the form of Se supplementation. This suggests that the effect of certain forms of Se on lipid oxidation becomes detectable during storage, while in fresh meat, the differences between treatments were not marked. Overall, nano-Se supplementation did not show a consistent improvement in lipid oxidative stability across the analyzed muscles. However, available data on the effects of nano-Se on muscle oxidative stability in weaned piglets remain limited. The differences in TBARS between treatments may be partially related to the chemical form of Se. However, this interpretation should be made with caution, as the response was inconsistent across muscles. The organic form of Se is more efficiently deposited in muscle tissue and participates in the synthesis of selenoproteins [44]. Under storage conditions, this may result in lower TBARS values. Hernández-García et al. [10] observed a similar effect, with Se-biofortified feeds improving the oxidative stability of pork meat and reducing *post-mortem* lipid oxidation during storage. Miqueletto et al. [64] also investigated the effect of supplementation with organic forms of Se on pork meat stability during storage. They concluded that storage duration was the main factor affecting TBARS values, while the addition of Se to the diet had no significant effect. Compared to organic Se, nano-Se must be converted to selenide before it can be incorporated into selenoproteins, which affects its concentration in muscle tissue [44,52]. Although it may promote antioxidant activity in vivo, it may not consistently protect lipids during storage, which may explain differences in TBARS dynamics between Se sources.

Different forms of Se can affect the oxidative stability of individual muscles, especially during storage. However, storage time remains the main factor determining the increase in TBARS values in weaned piglet tissue.

## 5. Conclusions

The results of this study show that the post-weaning period strongly influences hematological and antioxidant parameters in weaned piglets. These changes are largely associated with age and post-weaning stress. The addition of Se to the post-weaning piglet diet affected MCHC and PLT levels, while other hematological parameters were unaffected by Se supplementation. The formulated Se treatments modulated antioxidant enzyme activity and protein oxidation-related parameters of piglets, as shown by changes in plasma and erythrocyte glutathione peroxidase activity and carbonyl protein levels. In our study, nano-Se showed a relatively consistent temporal pattern of antioxidant enzyme activity. Analysis of lipid oxidation in muscle tissue showed that storage time was a key factor in increasing TBARS values. In contrast, the effect of Se supplementation was limited and depended on muscle type.

The results indicate that Se supplementation at the formulated nutritional level has a limited but measurable effect on antioxidant enzyme-related parameters, while changes in hematological indicators are primarily related to physiological adaptation to the post-weaning period.

## Figures and Tables

**Table 1:** Analyzed chemical composition and estimated metabolizable energy of the basal diet.

**Component (%)**	**%**
Corn	45.00
Barley	10.00
Wheat feed flour	5.00
Soybean meal	15.00
Full-fat soy	10.00
Dry beetroot noodles	3.00
Milk substitute	5.00
Yeast	1.00
Methionine	0.50
Lysine	1.00
Dextrose	0.50
Sea salt	0.40
Calcite flour	0.30
Monocalcium phosphate	0.30
Premix	3.00
**Basic Chemical Composition**	**%**
Dry matter	85.00
Crude protein	20.00
Crude fiber	4.00
Crude fat	3.00
Metabolizable energy (MJ/kg)	13.5

In the basal diet, chemical analysis determined 0.11176 mg Se/kg in the control diet (K). The analyzed Se concentrations in the final feed mixtures were 0.11187 mg/kg in P1, 0.23546 mg/kg in P2, and 0.10153 mg/kg in P3. The P1, P2, and P3 diets were formulated to provide the same target Se concentration of 0.3 mg/kg from different selenium sources. Se in the experimental groups was present in different chemical forms.

**Table 2:** Effects of different dietary selenium sources on hematological parameters in weaned piglets sampled on days 0, 22, and 45 post-weaning.

Parameters	Treatments												SEM	*p*-Value		
	Control			Organic Se			Biofortified Se			Nano-Se						
Days	0	22	45	0	22	45	0	22	45	0	22	45		*p*_T_	*p*_D_	*p*_T_ × *p*_D_
WBC, ×10^9^/L	16.43 ^b^	23.08 ^a^	20.13 ^a^	13.40 ^b^	20.53 ^a^	19.54 ^a^	14.74 ^b^	22.58 ^a^	22.54 ^a^	16.90 ^b^	23.60 ^a^	16.92 ^b^	1.42	0.174	0.001	0.173
RBC, ×10^12^/L	5.56 ^c^	5.82 ^b^	6.03 ^a^	5.70 ^b^	5.69 ^c^	5.81 ^a^	5.55 ^c^	5.77 ^b^	6.34 ^a^	5.43 ^c^	5.93 ^b^	5.98 ^a^	0.15	0.746	0.001	0.170
HGB, g/L	116.20 ^a^	101.89 ^b^	106.90 ^a^	117.20 ^a^	100.80 ^b^	100.30 ^b^	118.30 ^a^	103.90 ^b^	110.70 ^b^	111.90 ^a^	103.40 ^b^	102.30 ^b^	2.94	0.202	0.001	0.399
HCT, L/L	0.37 ^a^	0.35 ^b^	0.37 ^a^	0.38 ^a^	0.35 ^b^	0.35 ^b^	0.38 ^a^	0.35 ^b^	0.38 ^a^	0.37 ^a^	0.36 ^b^	0.37 ^a^	0.01	0.4997	0.005	0.451
MCV, fL	66.89 ^a^	60.37 ^b^	61.57 ^b^	66.34 ^a^	61.05 ^b^	60.64 ^b^	68.32 ^a^	61.20 ^b^	59.84 ^b^	67.99 ^a^	60.51 ^b^	59.61 ^b^	0.92	0.910	0.001	0.325
MCH, pg	20.93 ^a^	17.54 ^b^	17.70 ^b^	20.57 ^a^	17.71 ^b^	17.29 ^b^	21.43 ^a^	17.98 ^b^	17.47 ^b^	20.71 ^a^	17.42 ^b^	17.12 ^b^	0.36	0.296	0.001	0.792
MCHC, g/L	312.50 ^aAB^	290.89 ^bAB^	287.00 ^bAB^	310.20 ^aAB^	290.50 ^bAB^	284.70 ^bAB^	313.20 ^aA^	294.20 ^bA^	291.50 ^bA^	304.40 ^aB^	288.30 ^bB^	286.90 ^bB^	2.43	0.029	0.001	0.694
PLT, ×10^9^/L	422.20 ^B^	461.11 ^B^	433.90 ^B^	516.60 ^AB^	494.50 ^AB^	391.40 ^AB^	427.60 ^AB^	509.40 ^AB^	475.60 ^AB^	555.90 ^A^	562.20 ^A^	527.50 ^A^	41.66	0.049	0.175	0.476

Control = basal diet without Se supplementation; Organic Se = basal diet + organic Se; Biofortified Se = basal diet + Se from biofortified feed; Nano-Se = basal diet + nano-Se. WBC = total white blood cell count; RBC = red blood cell count; HGB = hemoglobin concentration; HCT = hematocrit; MCV = mean corpuscular volume; MCH = mean corpuscular hemoglobin; MCHC = mean corpuscular hemoglobin concentration; PLT = platelet count. *p*_T_ = *p* treatment; *p*_D_ = *p* day; *p*_T_ × *p*_D_ = *p* value for interaction between treatments and days post weaning. ^a,b,c^—Different lowercase superscripts indicate significant differences among sampling days within the same treatment (*p* < 0.05). ^A,B^—Different uppercase superscripts indicate significant overall treatment differences for parameters with a significant treatment effect and no treatment × sampling time interaction (*p* < 0.05).

**Table 3:** Effects of different dietary selenium sources on the antioxidant enzyme activities and protein oxidation in weaned piglets sampled on days 0, 22, and 45 post-weaning.

Parameters	Treatments												SEM	*p*-Value		
	Control			Organic Se			Biofortified Se			Nano-Se						
Days	0	22	45	0	22	45	0	22	45	0	22	45		*p*_T_	*p*_D_	*p*_T_ × *p*_D_
GPX_P_, U/L	363.55 ^aB^	299.44 ^bB^	508.57 ^aA^	412.85 ^aAB^	313.21 ^bAB^	244.01 ^bB^	437.40 ^aA^	373.19 ^bA^	284.79 ^bAB^	366.33 ^aB^	270.73 ^bB^	267.91 ^bB^	297.26	0.003	0.001	0.001
GPX_E_, U/mg	74,835.00 ^aA^	71,259.46 ^aA^	59,574.26 ^bA^	58,199.78 ^aB^	63,831.82 ^aB^	38,313.64 ^bB^	72,518.9 ^aA^	71,067.78 ^aA^	44,983.18 ^bA^	72,515.18 ^aA^	81,217.84 ^aA^	50,576.88 ^bA^	453.98	0.001	0.001	0.485
SOD_E_, µg/L	494.1 ^a^	416.78 ^b^	346.38 ^c^	456.5 ^a^	468.82 ^a^	379.4 ^b^	491.06 ^a^	427.62 ^b^	383.32 ^c^	441.62 ^a^	442.24 ^a^	352.34 ^b^	15.72	0.238	0.001	0.038
Catalase, U/mL	4814.54 ^a^	4235.66 ^a^	89.02 ^bB^	4577.04 ^a^	4355.09 ^a^	117.22 ^bA^	4763.18 ^a^	5050.38 ^a^	88.92 ^bB^	4692.07 ^a^	4496.47 ^a^	105.76 ^bA^	142.33	0.085	0.001	0.046
Carbonyls, nmol CP/mg protein	1.8 ^cA^	9.43 ^bA^	20.74 ^aB^	1.77 ^cA^	10.53 ^bA^	26.23 ^aAB^	1.61 ^cA^	10.34 ^bA^	25.16 ^aA^	1.05 ^cA^	9.85 ^bA^	22.66 ^aA^	0.63	0.001	0.001	0.001

Control = basal diet without Se supplementation; Organic Se = basal diet + organic Se; Biofortified Se = basal diet + Se from biofortified feed; Nano-Se = basal diet + nano-Se. GPX_P_ = Glutathione peroxidase activity in plasma; GPX_E_ = Glutathione peroxidase activity in erythrocytes; SOD_E_ = Superoxide dismutase activity in erythrocytes. *p*_T_ = *p* treatment; *p*_D_ = *p* day; *p*_T_ × *p*_D_ = *p* value for interaction between treatments and days post weaning. ^a,b,c^—Different lowercase superscripts indicate significant differences among sampling days within the same treatment (*p* < 0.05). ^A,B^—Different uppercase superscripts indicate significant differences among treatments within the same sampling day (*p* < 0.05).

**Table 4:** Effects of different dietary selenium sources on the TBARS of weaned piglets in different muscles.

	Treatments								SEM	*p*-Value		
	Control		Organic Se		Biofortified Se		Nano-Se			*p*_T_	*p*_D_	*p*_T_ × *p*_D_
Tissue	DAY 0	DAY 7	DAY 0	DAY 7	DAY 0	DAY 7	DAY 0	DAY 7				
TB, μg MDA/g	0.612 ^a^	1.191 ^a^	0.698 ^a^	1.074 ^a^	0.348 ^b^	1.739 ^a^	0.649 ^b^	1.174 ^a^	0.077	0.048	0.001	0.506
MLD, μg MDA/g	0.525 ^a^	1.986 ^a^	0.493 ^a^	1.154 ^a^	0.438 ^b^	1.542 ^b^	0.485 ^b^	1.609 ^b^	0.191	0.240	0.001	0.314
MIS, μg MDA/g	0.640 ^a^	1.672 ^a^	0.709 ^a^	0.920 ^a^	0.669 ^ab^	0.836 ^b^	0.609 ^ab^	1.133 ^a^	0.107	0.051	0.001	0.022

Control = basal diet without Se supplementation; Organic Se = basal diet + organic Se; Biofortified Se = basal diet + Se from biofortified feed; Nano-Se = basal diet + nano-Se. TB = *m. triceps femoris*; MLD = *m. longissimus dorsi*; MIS = *m. intercostales sternales*. *p*_T_ = *p* treatment; *p*_D_ = *p* day; *p*_T_ × *p*_D_= *p* value for interaction between treatments and storage time. ^a,b^—Different lowercase superscripts indicate significant differences between storage times within the same treatment (*p* < 0.05).

## Data Availability

The data presented in this study are available from the corresponding author upon reasonable request.

## Supplementary Materials

The following supporting information can be downloaded at: https://www.mdpi.com/article/10.3390/ani16111700/s1, Table S1: Body weight and average daily gain of piglets supplemented with different dietary selenium source.
