# Supplementary material for: Effects of Different Dietary Selenium Sources on Antioxidant Enzyme Activities and Selected Hematological Parameters in Weaned Piglets
Source: Animals (Basel). 2026 Jun 1;16(11):1700. doi: 10.3390/ani16111700 (PMC13255605; doi:10.3390/ani16111700)
Supplement: Supplementary file 1 [file animals-16-01700-s001.zip › animals-4333895-supplementary.pdf]

Table S1. Body weight and average daily gain of piglets supplemented with different dietary selenium source.

Parameters	Treatments												p-value		
	Control			Organic Se			Biofortified Se			Nano-Se			pT	pD	pT × pD
	0	22	45	0	22	45	0	22	45	0	22	45			
Body weight, kg	6.45 ± 0.12	11.85 ± 0.34	21.57 ± 0.85	6.40 ± 0.12	11.99 ± 0.41	20.80 ± 0.48	6.60 ± 0.12	12.65 ± 0.93	21.03 ± 1.62	6.40 ± 0.10	12.07 ± 0.48	21.41 ± 1.04	0.999	0.856	0.801
ADG, kg/day	0.25 ± 0.01	0.49 ± 0.02	0.37 ± 0.01	0.26 ± 0.01	0.45 ± 0.02	0.35 ± 0.01	0.25 ± 0.02	0.47 ± 0.02	0.36 ± 0.02	0.25 ± 0.01	0.49 ± 0.02	0.36 ± 0.01	0.913	0.254	0.106

Control = basal diet without Se supplementation; Organic Se = basal diet + organic Se; Biofortified Se = basal diet + Se from biofortified feed; Nano-Se = basal diet + nano-Se. ADG = average daily gain. Values are presented as mean ± SEM. p_T= *p* treatment; p_D= *p* day; p_T x p_D= p value for interaction between treatments and days post weaning.
